# A novel antibody treatment reduces deformed wing virus loads in the western honey bee (*Apis mellifera*)

**DOI:** 10.1128/msphere.00497-24

**Published:** 2024-10-30

**Authors:** N. J. J. MacMillan, B. M. Hause, T. Nordseth, A. Felden, J. W. Baty, J. L. Pitman, P. J. Lester

**Affiliations:** 1 School of Biological Sciences, Victoria University of Wellington, Wellington, New Zealand; 2 Cambridge Technologies, Worthington, Minnesota, USA; College of Agriculture, Life Sciences, and Natural Resources, University of Wyoming, Laramie, Wyoming, USA

**Keywords:** DWV, antibodies, IgY, honey bee, anti-viral, bioavailability

## Abstract

The deformed wing virus (*Iflavirus aladeformis*) (DWV) is a key driver of colony loss in the western honey bee (*Apis mellifera*). Here, we demonstrate that orally delivered anti-DWV antibodies can act systemically to reduce DWV loads in naturally infected honey bees. Immunoglobulin Y (IgY) was produced in adult chickens against two DWV proteins, harvested from their eggs, and fed to bees in a sucrose solution. An enzyme-linked immunosorbent assay demonstrated that orally delivered anti-DWV IgY migrated to the hemolymph. We next assessed the ability of orally delivered anti-DWV IgY to reduce DWV viral loads in naturally infected bees using qPCR. An antibody treatment resulted in a significant eightfold viral load reduction in DWV-infected bees. Our findings demonstrate the potential for antibody treatments to help mitigate the losses attributed to DWV in *A. mellifera*.

**IMPORTANCE:**

Deformed wing virus (DWV) is considered to be a key component of declining honey bee health which threatens global food production. The virus can result in significantly shortened lifespan, deformities in developing bees, and impaired cognition. There is currently no method to directly control the virus. The virus can be indirectly controlled with acaricidal treatments that target a key vector, the parasitic varroa mite (*Varroa destructor*). But acaricide resistance and a lack of effective alternatives for the control of both Varroa and DWV are major threats to beekeeping and the wider agricultural industry. Our research presents a significant development in the ability to reduce DWV burden in honey bees using IgY antibodies. Moreover, immunoglobulin Y has the potential to be more broadly established as a new treatment modality to combat other pathogens and parasites in *A. mellifera.*

## INTRODUCTION

The western honey bee (*Apis mellifera*) is facing high rates of colony loss globally that threatens both the apicultural industry (1–3) and pollination services necessary for food production (4). *Varroa destructor,* an obligatory ectoparasite of *A. mellifera,* is considered to be the main culprit in many of the honey bee colony losses worldwide (5, 6). A major component of the damage caused by Varroa is thought to be its association with deformed wing virus (DWV) (*Iflavirus aladeformis*), a positive single-stranded RNA virus in the family Iflavaridae (7–9). The inability of *A. mellifera* to tolerate Varroa infestations may even represent an intolerance to DWV rather than to the parasite itself (8). Overt DWV infections within individuals are characterized by deformed wings, shortened abdomens, reduced lifespan, and behavioral abnormalities, which can ultimately result in colony collapse (7, 10). The icosahedral capsid of the virus is non-enveloped with a diameter of 30 nm and is comprised of three major structural proteins VP1, VP2, and VP3 with masses 44, 32, and 28 kDa, respectively (9, 11).

Varroa infestations are typically controlled with acaricidal treatments, which can indirectly reduce DWV levels within treated colonies (12). However, acaricide-resistant Varroa populations have been found in Europe and North America in the 1990s and have since been identified globally (12–15). As a result, both Varroa and DWV are becoming a more prevalent problem (5, 6, 15).

Antibodies are a promising alternative to acaricides by directly treating DWV infections in *A. mellifera*. Antibodies can function independently from a cell-mediated immune response by binding to specific antigens and blocking their biological functions (16). This independence allows antibodies to be isolated and therapeutically transferred between individuals to treat disease (17, 18). These passive antibody therapies for systemic disease treatment in mammals require intravenous delivery as oral administration exhibits very poor to no bioavailability (19). It is unknown whether ingested antibodies can migrate across the gut wall and into the hemolymph in *A. mellifera*; however, the bioavailability of large proteins, including antibodies, has been seen in other insect species (20–22). Encouragingly, orally delivered IgY raised against the Chinese sacbrood virus (CSBV) protected the Asian, or eastern, honey bee (*Apis cerana*) against the virus (23). The elimination of CSBV from treated hives provides promising evidence for IgY as a treatment modality for honey bee viruses and suggests that antibodies may be orally bioavailable (23).

Antibodies have previously been raised against recombinantly expressed DWV virus capsid proteins (24–26). Wu et al. (25) expressed a truncated VP1 to isolate the P-domain for immunization in mice. Antibodies raised against the protein were used in tissue assays where they determined the P-domain to be important in the infection of cells but not host-cell entry (25). Fei et al. (26) expressed full-length VP1, VP2, and VP3 from which, antibodies were raised in mice and demonstrated *in vitro* neutralization (26). As of yet, the delivery and *in vivo* antiviral effect of therapeutic antibodies against DWV have not been investigated in DWV-infected bees.

Pathogen-specific immunoglobulin Y (IgY) is commercially produced and fed to livestock to reduce pathogen burden, in part due to its low production cost when compared to the more commonly used immunoglobulin G (IgG) (27, 28). In addition, unlike IgG that is collected from the blood of immunized mammals, IgY can be procured from the egg yolk of immunized hens (29). IgY is typically high yielding with the yolks containing two times the antibody concentration when compared to levels of IgG in the sera of mammals (30).

The global apicultural and agricultural industries need safe and effective DWV control options. We developed IgY antibodies against recombinant DWV capsid proteins. We then investigated the bioavailability of an anti-DWV IgY treatment using an enzyme-linked immunosorbent assay (ELISA) and found that IgY passed across the gut wall of adult bees and entered the hemolymph. Using qPCR, we showed that anti-DWV IgY treatment could decrease DWV loads in naturally infected adult bees.

## MATERIALS AND METHODS

### DWV antigen and anti-DWV IgY production

The DWV-A capsid proteins VP1 and VP3 were expressed in Sf9 cells using a baculovirus expression system as hexahistidine-tagged fusion proteins and purified by affinity chromatography by Genscript (USA). VP2 had difficulty expressing in sufficient quantities, as such VP2 was not included in the immunization preparations. The DWV immunization was prepared with 10 µg of each of the recombinant DWV proteins and 67% (vol/vol) incomplete Freund’s adjuvant in 1 mL of 0.1 M phosphate-buffered saline (PBS). To produce the anti-DWV IgY, white leghorn hens (*Gallus gallus domesticus*) were immunized three times at 21-day intervals. Eggs were collected daily 2 weeks after the final immunization and the IgY was purified from the yolks (31, 32). The handling and immunization of hens were performed in accordance with the Institutional Animal Care and Use Committee, under approval number TGB19001.

The final IgY concentration was determined with a sandwich ELISA. Wells within a 96-well plate were coated with 200 µL of a 1:2,000 dilution of 4.5 mg/mL goat anti-chicken IgG in 0.05M carbonate-bicarbonate buffer and incubated overnight at 4°C. Wells were then blocked with 210 µL of 5% (wt/vol) skim milk powder (SMP) in carbonate-bicarbonate buffer and incubated for 1 h at 37°C. After blocking, wells were washed three times with 210 µL of wash buffer [0.01 M (PBS) and 0.005% (vol/vol) Tween20]. About 200 µL of 1.0 mg/mL IgY standard at twofold dilutions of 1:800 through 1:51,200 and the anti-DWV IgY sample at the same dilutions in assay buffer [5% (wt/vol) SMP in PBS-Tween20] were loaded into wells. The plates were incubated at 37°C for 2 h and washed three times with 210 µL of wash buffer. Plates were then incubated at 37°C after the addition of a 1:2,000 dilution of 1.0 mg/mL goat anti-chicken horseradish peroxidase conjugate in assay buffer for 1 h and washed three times with 210 µL of wash buffer. After washing, 100 µL of 0.1 mg/mL o-phenylenediamine dihydrochloride and 0.004% (vol/vol) hydrogen peroxide in 0.05 M phosphate-citrate buffer were added. Plates were incubated at 37°C for 30 min, the reaction was stopped with the addition of 50 µL of 2.5 M HCl, and the absorbances were measured at 492 nm on a SPECTROstar Nano plate reader (BMG LABTEC, Germany). The binding absorbance/binding absorbance max (*B*/*B*_max_) was calculated, following the correction for non-specific binding. The final IgY concentration was determined by comparing the sample *B*/*B*_max_ to that of the linear component of the standard curve.

### DWV specificity of anti-DWV IgY

The specificity of the IgY to native VP1 and VP3 was assessed using a western blot. Ten bees with deformed wings were collected from the frames of a single hive with a known Varroa infestation at Victoria University of Wellington (VUW), New Zealand. The bees were homogenized in a Precellys Evolution homogenizer (Bertin Instruments, France) for three cycles of 20 s at 4,500 rpm. Total protein was extracted from 100 mg of the homogenate with 990 µL RIPA lysis buffer and 10 µL protease inhibitor cocktail. The homogenate was incubated on ice for 20 min before centrifugation at 13,000 × *g* for 20 min at 4°C. About 10 µL of the supernatant was heated at 95°C for 5 min in SDS before loading on a 4–20% SDS-PAGE gel. The gel was run at 50 V for 30 min then 120 V for an hour. Protein was then transferred to a PVDF membrane (prewetted with methanol) at 100 V for 1 h in transfer buffer [3% glycine (wt/vol), 14.5% tris base (wt/vol), and 20% methanol (vol/vol)]. The membrane was blocked for an hour with 5% SMP (wt/vol) in TBS-T [2.5% tris base (wt/vol), 8.8% NaCl (wt/vol), and 0.1% Tween20 (vol/vol) (pH 7.6)] and then washed with TBS-T. The membrane was incubated with a 1:1,000 dilution of the anti-DWV IgY in 5% SMP (wt/vol) TBS-T for 16 h at 4°C. The membrane was washed with TBS-T before being incubated with a 1:2,000 dilution of 1.0 mg/mL goat anti-chicken horseradish peroxidase conjugate in 5% SMP (wt/vol) TBS-T at room temperature for 2 h. The membrane was washed a final time with TBS-T, and developed for 5 min with enhanced chemiluminescence before being imaged with an Omega Lum G Imaging System (Gel Company, USA).

### Relative immunodominance of VP1 and VP3

The relative immunodominance of VP1 and VP3 in stimulating IgY production in hens was determined with an ELISA as previously described but with the following modifications. Wells of a 96-well plate were coated with VP1 (1 µg/mL) or VP3 (1 µg/mL) separately in 0.05 M carbonate-bicarbonate buffer and incubated overnight at 4°C. Wells were blocked and washed as previously described. About 200 µL of anti-DWV IgY in assay buffer was loaded into wells coated with either VP1 or VP3 as a twofold dilution series from 1:200 to 1:25,600. The relative immunodominance of VP1 and VP3 was determined from the *B*/*B*_max_ values for the given anti-DWV IgY dilutions where a linear relationship was shared between the antigens.

### Bioavailability of IgY in adult honey bees

Adult *A. mellifera* were treated with IgY in the laboratory to investigate whether the antibodies exhibited oral bioavailability. Live adult nurse bees were collected from a single hive with a known Varroa infestation at VUW. The collected bees were anesthetized by chilling and then placed into rearing containers (65 × 65 × 105 mm^3^) (33). Each rearing container housed 20 bees. The bees were either supplied with 2 mL of the IgY treatment [300 ng/mL IgY in 50% (wt/vol) sucrose, 2% (wt/vol) honey] (*n* = 5) or 2 mL of the no IgY control [50% (wt/vol) sucrose, 2% (wt/vol) honey] (*n* = 5). All bees were supplied with 3 mL water. The rearing containers were kept in a climate-controlled room at 34°C and 60% relative humidity (RH). Water and feed were replaced daily.

After 24 h (day 1) and 72 h (day 3), all bees were euthanized and kept on ice during the hemolymph extraction and dissection procedure. The hemolymph was extracted from bees by carefully separating the head from the thorax while leaving the esophagus attached. The subsequent bead of hemolymph was then aspirated with a glass capillary tube and the volume was recorded. Hemolymph was pooled from five bees per replicate for the control and 10 per replicate for the treated bees in order to make paired samples for contamination control analysis. The pooled hemolymph of treated and control bees was dispensed into 1.5 mL tubes containing 400 µL or 200 µL of assay buffer (5% wt/vol skim milk powder in 0.1 M PBS), respectively. The digestive tracts of the treated and control bees were then removed and placed into reinforced homogenization tubes with 500 µL or 250 µL of assay buffer, respectively. Gut samples were homogenized in a Precellys Evolution homogenizer (Bertin Instruments, France) for two cycles of 20 s at 4,500 rpm. Hemolymph and gut samples from IgY-treated groups were vortexed, and aliquoted into two tubes to create paired samples for internal contamination analysis. The processed samples were stored at −20°C.

To verify that the hemolymph extraction method did not result in hemolymph becoming contaminated with IgY from the exterior of the bee, experiments were conducted whereby hemolymph was extracted from untreated bees externally contaminated with the IgY treatment feed. Bees were collected from the entrances of hives and euthanized by chilling. Five bees were placed into 15 mL tubes containing 50 µL of the 300 ng/µL IgY treatment feed (*n* = 5). The bees were gently rolled in the feed for 3 min before the hemolymph was extracted, as described above.

To estimate the IgY recovery of the ELISA for titer corrections, the hemolymph (*n* = 5) and guts (*n* = 5) were extracted from bees fed with a sucrose/honey control. Hemolymph samples were then spiked with IgY to a final in-assay dilution factor of 1:102,400.

An ELISA was used to determine the bioavailability of IgY in adult bees fed with IgY for 1 and 3 days compared to that of controls. The ELISA was performed as previously described. The *B*/*B*_max_ values of both the digestive tract and hemolymph samples were compared between IgY-fed and control bees. Analyses were performed with a Kruskal-Wallis test in *R* (version 4.2.2) (34). ELISAs for the detection of potential hemolymph contamination from the exterior of the bee, and estimations of IgY recovery from hemolymph and gut samples spiked with a known IgY dilution were performed. Finally, hemolymph IgY concentrations were determined against the IgY standard and corrected by the recovery determined from IgY spiked hemolymph and normalized to the volume of extracted hemolymph.

To identify IgY contamination because of potential damage to the digestive tract during hemolymph extraction, immune-labeled Dynabeads were fed to the day 1 IgY-treated bees prior to hemolymph extraction. At day 1, bees were anesthetized by chilling after 69 h of IgY treatment. Next, bees were placed into harnesses as described by Williams et al. (33) and were kept at 34°C and 60% RH for 1 h to regain consciousness (33). Bees (*n* = 5) were individually fed 10 µL of the internal contamination control feed containing 10% (vol/vol) anti-sheep rabbit IgG-labeled Dynabeads in a 50% (wt/vol) sucrose, 2% (wt/vol) honey solution. The immune-labeled magnetic beads were monosized with a diameter of 2.8 µm (CV <5%), and as such, would not be able to pass across the gut wall. The bees were then released back into the rearing container for 1 h at 34°C and 60% RH to await the extraction and dissection procedure as described above to produce paired day 1 samples for IgY and immune-labeled bead detection.

To identify samples in which the digestive tract was damaged during hemolymph extraction, potentially resulting in internalized IgY contaminating the hemolymph, magnetic separation, and fluorophore labeling were used to detect the immune-labeled magnetic beads fed to bees. The detection of beads would be indicative of contamination. Hemolymph and digestive tract samples from bees treated with IgY and immune-labeled Dynabeads underwent magnetic pull down for 3 min. Samples were washed two times with PBS. About 5 µL of the fluorophore-antibody solution [5% (wt/vol) SMP, 9% (vol/vol) rabbit anti-sheep Alexa 680 fluorophore conjugate, in PBS] was added to the tubes and incubated in the dark at room temperature for 15 min. Tubes were then placed on a magnetic rack for 3 min and washed with PBS four times. Samples were resuspended in 10 µL of PBS and loaded onto a hemacytometer for fluorescent imaging. Images were processed with a custom *R* code that produced a count of the number of pixels above an intensity threshold (supplementary materials).

### Treatment of naturally DWV-infected adult bees with anti-DWV IgY

Naturally, DWV-infected adult *A. mellifera* were orally treated with anti-DWV IgY to ascertain the effect on DWV load. Adult nurse bees were collected from the frames of three symptomatic hives on the university campus and transferred to the rearing containers in the lab. Each rearing container contained 15 bees and was supplied with either: (i) a low-dose treatment [5 ng/µL anti-DWV IgY in 30% (wt/vol) sucrose water] (*n* = 14); (ii) a high-dose treatment [50 ng/µL anti-DWV IgY in 30% (wt/vol) sucrose water] (*n* = 9); and (iii) a vehicle only control [30% (w/v) sucrose water] (*n* = 15). Bees were left to fed *ad libitum* with the treatments and water being replaced daily. After 7 days, bees were euthanized with CO_2_, snap-frozen on dry ice, and stored at −80°C.

The samples taken of bees from the control, high, and low IgY treatments were processed for qPCR analysis of DWV viral loads. Six bees from each replicate were pooled and homogenized by bead beating at 4,500 rpm for 30 s in a Precellys homogenizer and immediately placed on dry ice. A chloroform/phenol RNA extraction protocol was used on the homogenate (35). The concentrations and purities of the extracted RNA were measured with an Implen NanoPhotometer (Germany). Genomic DNA was digested using the Thermofisher Exonuclease I kit (ThermoFisher Scientific, USA) as per the manufacturer’s instructions. Sample cDNA was synthesized using qScript cDNA SuperMix (Quanta Biosciences, USA) or SuperScript IV RT (ThermoFisher Scientific, USA) following manufacturer’s directions.

We used qPCR to determine the DWV loads. Each 20 µL reaction contained 10 µL of PowerUP SYBR Green Master mix (Applied Biosystems, USA), 2 µL of 0.5 µM forward and reverse primers for the DWV-A genome (36) or for the *A. mellifera* reference gene, *Ndufa8* (37), and 8 µL of 1.25 ng/µL cDNA. No template and no reverse transcriptase controls were included in each qPCR run. The qPCR protocol was as follows: 50°C for 2 min, 95°C for 2 min followed by 40 cycles of 95°C for 15 s, 59°C for 15 s, and 72°C for 1 min. Normalized viral loads were calculated using the 2^−ΔΔCq^ method (38). Differences in the 2^− ΔΔCq^ values between treatment groups were analyzed with a Kruskal-Wallis test followed by the Dunn *post hoc* test using the Benjamini Hochberg procedure using the *FSA* package (39) in R (34).

## RESULTS

**Fig 1:**
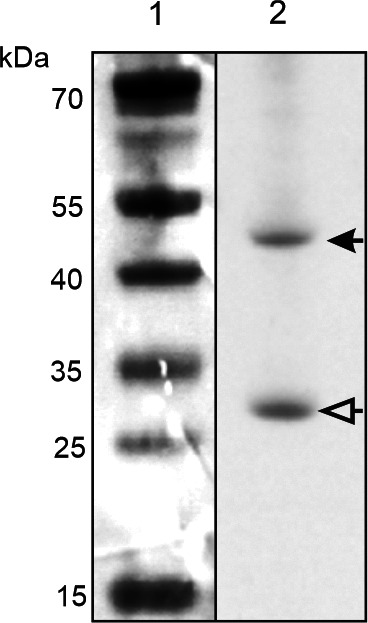
Specificity of anti-DWV IgY against native DWV capsid proteins VP1 and VP3. Lane 1 contains a protein standard ladder. Lane 2 contains protein extracted from honey bees infected with DWV. The black arrow indicates VP1 (~44 kDa), and the white arrow indicates VP3 (~28 kDa).

We first determined total IgY concentrations, native VP1 and VP3 specificity, and relative VP1 and VP3 immunodominance. The total IgY concentration of the purified yolk was 1.2 mg/mL. The anti-DWV IgY generated with recombinant VP1 and VP3 showed specificity to the native VP1 and VP3 DWV capsid proteins in naturally infected bees (Fig. 1). VP1 and VP3 proportionally constituted 0.36 and 0.64 of the specific IgY, respectively (SE = 0.060).

**Fig 2:**
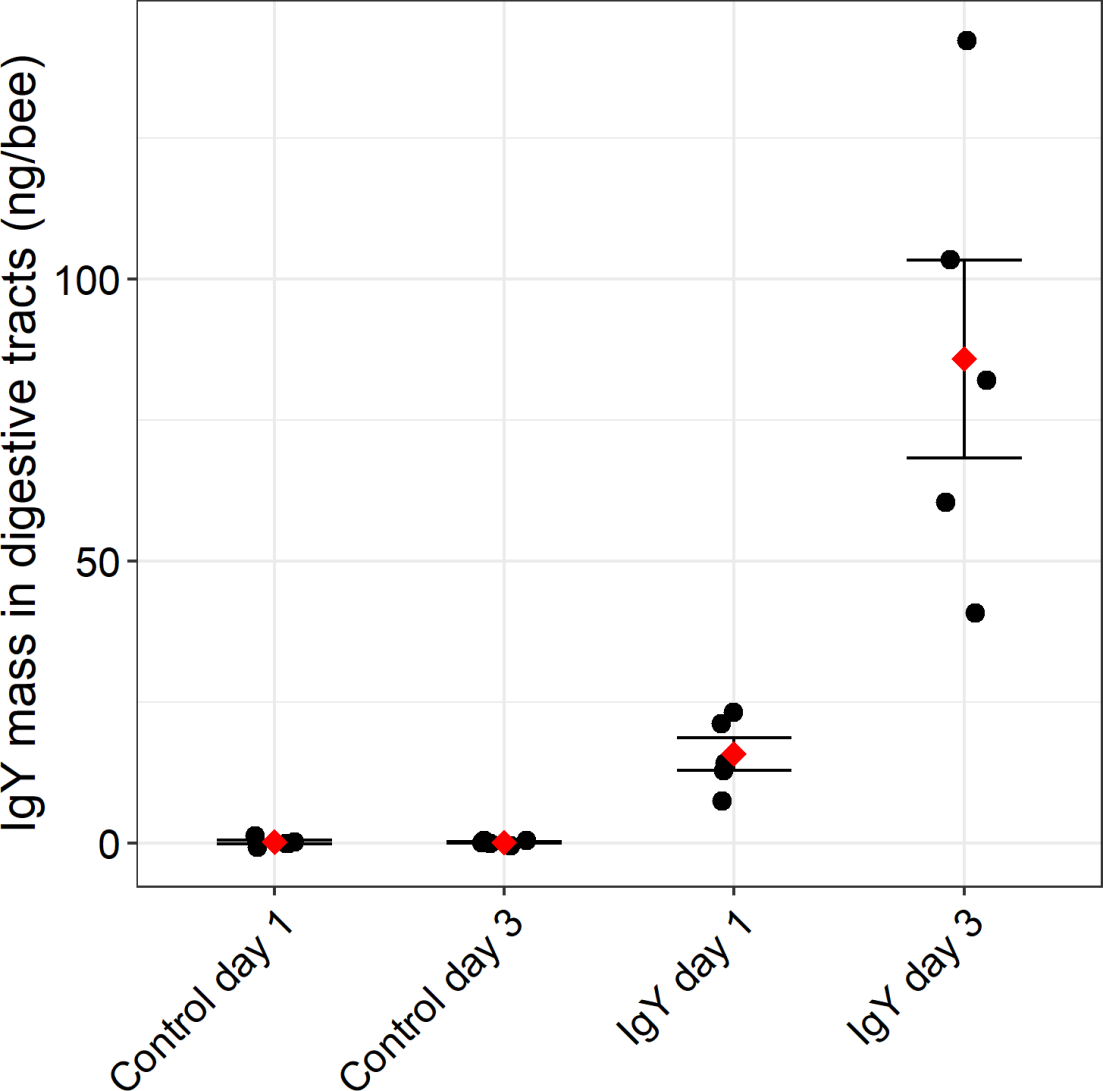
IgY mass within the digestive tract of bees after oral administration IgY for 1 and 3 days. Adult bees were orally treated with 300 ng/mL IgY in a 50% (wt/vol) sucrose 2% (wt/vol) honey solution or the control [50% (wt/vol) sucrose 2% (wt/vol) honey solution] for 24 h (day 1) and 72 h (day 3). The black dots indicate the IgY masses of the individual replicates and the red diamonds indicate the mean for each group. Error bars are ±standard error. Statistically significant levels of IgY were detected in the gut of day 3 bees (mean mass per bee = 85.8 ng ± 17.6 SE, *Z* = −3.208, *P* = 0.004).

**Fig 3:**
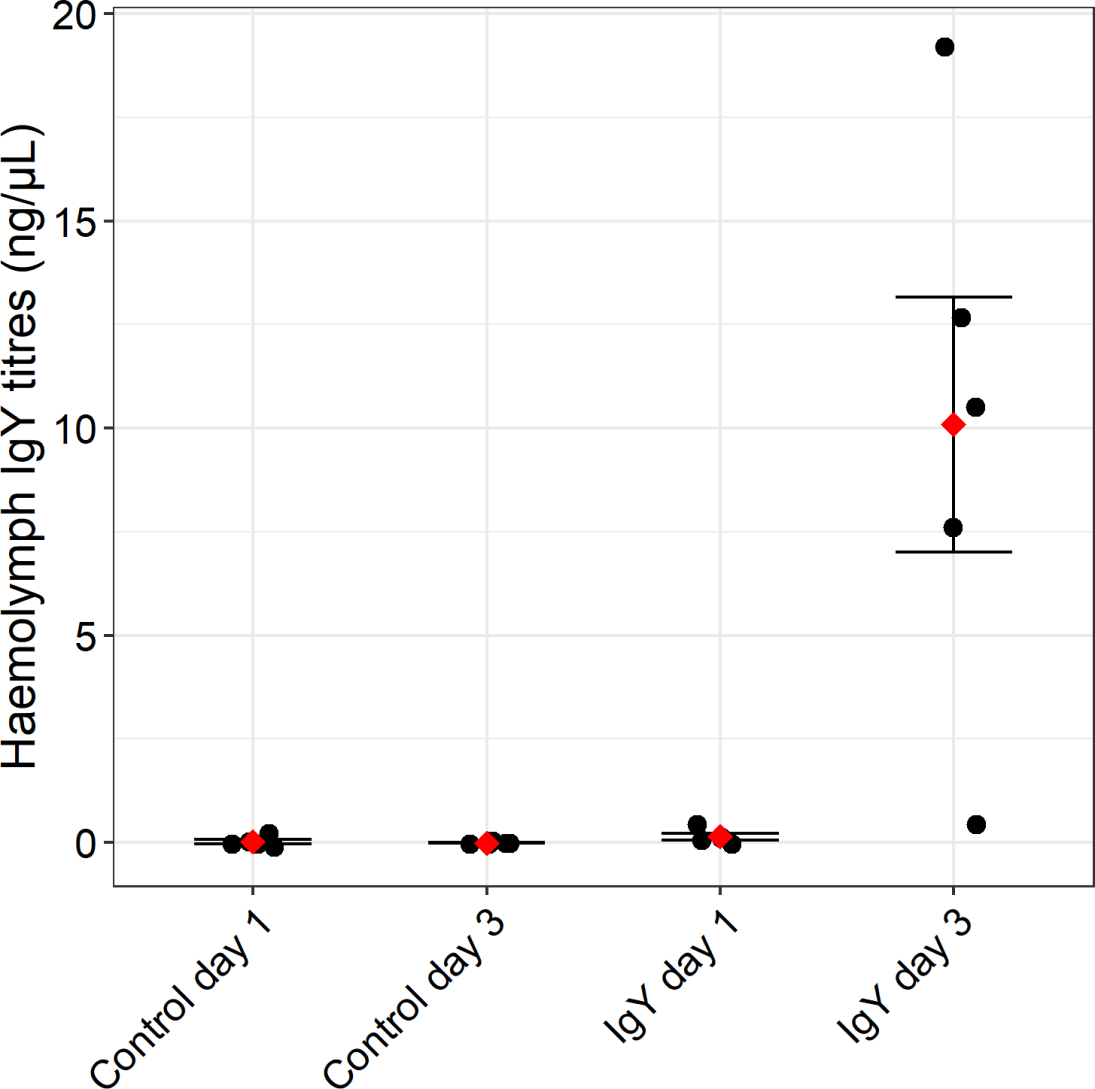
IgY concentrations in the hemolymph of bees after oral administration with IgY for 1 and 3 days. Adult bees were orally treated with 300 ng/mL IgY in a 50% (wt/vol) sucrose 2% (wt/vol) honey solution or the control [50% (wt/vol) sucrose 2% (wt/vol) honey solution] for 24 (day 1) and 72 h (day 3). The black dots indicate the hemolymph IgY concentration of the individual replicates and the red diamonds indicate the mean for each group. Error bars are ±standard error. Statistically significant levels of IgY were detected in the hemolymph of day 3 bees (mean concentration = 10.1 ng/µL ± 3.1 SE, *Z* = −3.315, *P* = 0.010).

Digestive tract and hemolymph samples were analyzed to ascertain the bioavailability of IgY in bees after oral treatment. IgY was detected in the guts of bees at day 1 (mean mass per bee = 15.9 ng ± 2.87 SE, *Z* = −2.139, *P* = 0.065) and day 3 (mean mass per bee = 85.8 ng ± 17.6 SE, *Z* = −3.208, *P* = 0.004) when compared to the corresponding no IgY controls (Fig. 2). IgY was not detected in the hemolymph in day 1 treated bees (mean concentration = 0.141 ng/µL ± 0.078 SE, *Z* = −1.230, *P* = 0.263; Fig. 3). However, IgY was detected in the hemolymph on day 3 at significant levels when compared to the corresponding control (mean concentration = 10.1 ng/µL ± 3.07 SE, *Z* = −3.315, *P* = 0.010).

**Fig 4:**
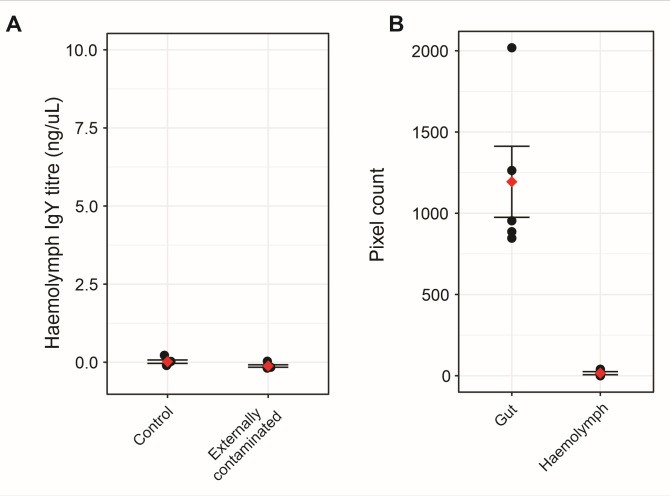
(**A**) IgY concentration in the hemolymph of bees externally contaminated with IgY and the no IgY contamination control. The black dots indicate the *B*/*B*_max_ of the individual replicates and the red diamonds indicate the mean for each group. Error bars are ±standard error. The mean IgY concentration of the externally contaminated bees is not significantly different from the control (mean IgY concentration = −0.005 ng/µL ± 0.002, *df* = 1, *χ*^2^ = 2.827, *P* = 0.093). (**B**) Pixel count indicative of amount of immune-labeled magnetic beads in the gut and hemolymph of bees of day 3 IgY-treated bees fed 10% (vol/vol) anti-sheep rabbit IgG labeled Dynabeads in a 50% (wt/vol) sucrose 2% (wt/vol) honey solution. Black dots indicate the pixel counts of individual replicates and the red diamonds indicate the mean. Error bars are ±standard error. Two of the five treated replicates contained low levels of magnetic beads.

To validate that IgY detected in the hemolymph was due to the antibody crossing the gut wall, we performed external and internal contamination controls. IgY was not detected in the hemolymph of externally contaminated bees (mean concentration = −0.121 ng/µL ± 0.039, *df* = 1, *χ*^2^ = 3.153, *P* = 0.0758) (Fig. 4A). The internal control identified potential hemolymph contamination from the gut in two of the five samples. When internalized contamination did occur, it was at very low levels (Fig. 4B). A correlation analysis of the paired IgY detection and internal contamination control samples showed that internalized contamination did not explain hemolymph IgY levels (Spearman’s rank correlation coefficient = −9.061e−05).

**Fig 5:**
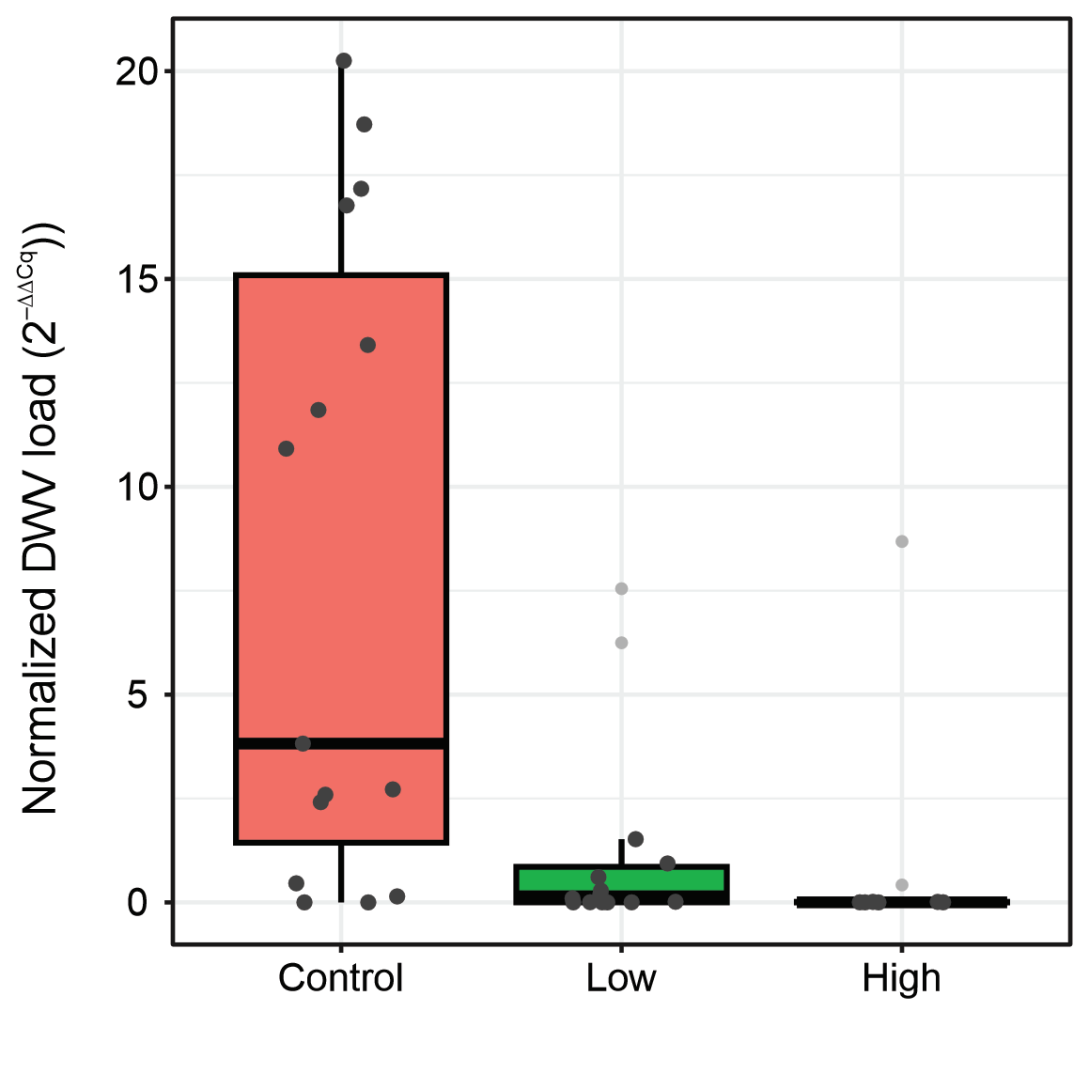
Normalized DWV loads in bees after an orally administered anti-DWV IgY treatment. Bees were orally administered either a 30% (wt/vol) sucrose water control, or low (5 ng/µL) and high (50 ng/µL) dosage anti-DWV IgY treatments. DWV was measured using qPCR following 7 days of treatment. Dots indicate the viral load in individual samples. Outliers are indicated by light gray dots. Normalized DWV loads significantly differed as a result of the oral anti-DWV IgY treatments for low (*Z* = 2.29, *P* = 0.033) and high doses (*Z* = 2.95, *P* = 0.001).

We next investigated how the oral administration of anti-DWV IgY affected DWV viral loads in bees. Nonsymptomatic bees were collected from Varroa-infested hives containing individuals displaying overt DWV infections. The bees were fed with either low or high doses of anti-DWV IgY or the sugar water control. Using qPCR, we measured the DWV loads after 7 days of treatment. Median DWV loads were significantly different between the experimental groups (*df* = 2, *χ*^2^ = 10.02, *P* = 0.007). Median DWV loads decreased in relation to the control group by 3.8-fold in the low-dose treatment group (*Z* = 2.30, *P* = 0.033) and 8.5-fold in the high-dose treatment group (*Z* = 2.95, *P* = 0.010), while no significant difference was found between the low- and high-dose treatment groups (*Z* = −0.91, *P* = 0.361) (Fig. 5). The high degree of variation in DWV loads of the control group should be expected as some bees would have been parasitized by Varroa during their development, which would have resulted in high DWV loads (40), while other bees would have not.

## DISCUSSION

DWV represents an increasing global threat to the agricultural industry and the pollination services honey bees provide (2, 5, 6, 41, 42). An antibody treatment could offer a potentially highly targeted and environmentally friendly solution to viral infections. We developed anti-DWV IgY against a combination of two recombinant viral capsid proteins (VP1 and VP3). The resultant antibodies demonstrated specificity to native DWV VP1 and VP3. Of the two antigens, VP3 appeared to have elicited a stronger IgY response in the immunized hens, producing approximately two times the levels when compared to VP1 as seen with IgG levels in mice (26). As the immunization preparations were comprised of both VP1 and VP3, the protection that either antigen-specific antibody offered is unknown.

The feeding of adult bees with the anti-DWV IgY resulted in the IgY being detected in the hemolymph of honey bees. The variable IgY levels in the guts of treated bees suggest that the volume of treatment consumed by bees varies. This result was expected from an *ad libitum* feeding design due to the inter-individual food intake observed in bees (43). The IgY detected within the hemolymph is likely not from hemolymph contamination from the exterior or from damage to the digestive tract during extraction, but rather as a result of IgY crossing the gut wall and entering the hemolymph. Our finding that IgY is present in the hemolymph at day 3 and not at day 1 suggests that movement across the gut wall is relatively slow, supporting the hypothesis that the movement of large proteins across the gut wall of insects occurs via passive diffusion (20). Our results showing that IgY is bioavailable in bees indicate that antibodies could be an effective treatment modality for apicultural applications. It seems likely that the treatment will have a systemic distribution due to circulation within the hemolymph and that it will exhibit tissue penetrance, as seen with IgG in mammalian tissue (34). However, it is highly unlikely that the treatment is cell permeable (35). DWV has been shown to cause persistent infections in the AmE117 embryonic honey bee cell line without cytopathogenicity (44). Taken together, the inability for the antibody to access the intercellular environment and the persistence of DWV infections in honey bees, may mean lengthy treatment regimens would be needed for viral clearance.

We orally administered anti-DWV IgY at either a low- (5 ng/µL) or high- (50 ng/µL) dose in sugar water to naturally DWV-infected *A. mellifera* for 7 days. We observed significant ~3-fold and ~8-fold decreases in median DWV loads in bees that received the low- and high-dose anti-DWV IgY treatments, respectively. Our study has demonstrated the ability to directly control DWV in infected bees, which, to our knowledge, is the first time DWV infection has been controlled with an antibody treatment. Whether the treatment alone can improve colony health or if a combinatorial approach with acaricides would be required is still to be determined. Varroa-mediated vectoring of DWV to developing bees can result in overt infections (7). Therefore, investigating the potential of anti-DWV IgY on developing bees is a critical next step. The treatment could function as a prophylaxis for brood in the 4- to 5-day window before Varroa parasitism, and hence DWV vectoring, occurs in the late larval and pupal stages. Varroa and DWV appear to occur in a mutualistic relationship, with the viral pathogen exerting an immunosuppressant effect that enhances mite reproduction (45). It is thus possible that the treatment of Varroa-infested hives with anti-DWV IgY could limit the damage caused by Varroa.

Our findings show promise for antibodies as a novel DWV control treatment in *A. mellifera.* Our research also provides a framework in which a toolbox of IgY treatments could be developed for a range of bee parasites and pathogens. These possible targets could include the bacterial pathogen *Paenibacillus larvae*; the microsporidia *Nosema* spp.; and other damaging viruses such as the Israeli acute paralysis virus and Black queen cell virus. Furthermore, Varroa itself may be able to be targeted and controlled via the binding of essential mite proteins. Antibodies as a treatment modality could also be expanded to other economically significant insect species such as the disease-vulnerable silkworm (*Bombyx mori*). Our findings demonstrate the potential of IgY antibodies as a non-invasive, modular tool for the control of disease in honey bees.

## SUPPLEMENTAL MATERIAL

Supplemental material msphere.00497-24-s0001.docx R code for counting pixels for internal contamination analysis.

Legend msphere.00497-24-s0002.docx qPCR data set legend.

qPCR data set msphere.00497-24-s0003.csv Raw Ct values from the qPCR for both the DWV levels and the reference gene.

ASM does not own the copyrights to Supplemental Material that may be linked to, or accessed through, an article. The authors have granted ASM a non-exclusive, world-wide license to publish the Supplemental Material files. Please contact the corresponding author directly for reuse.
