# Supplementary material for: A novel antibody treatment reduces deformed wing virus loads in the western honey bee (Apis mellifera)
Source: mSphere. 2024 Oct 30;9(11):e00497-24. doi: 10.1128/msphere.00497-24 (PMC11580425; doi:10.1128/msphere.00497-24)
Supplement: Supplemental material — R code for counting pixels for internal contamination analysis. [file msphere.00497-24-s0001.docx]

Supplementary methods

R code for internal contamination analysis. The code counts the number of red pixels in an image of a haemolymph sample relating to the number of immunolabelled magnetic beads.

library(jpeg)

image_path1 <- ".jpg"

img1 <- readJPEG(image_path1)

brightness <- apply(img1, c(1, 2), function(x) sum(x) / 3)

brightness_threshold <- 0.3

bright_pixels <- brightness > brightness_threshold

bright_pixel_count <- sum(bright_pixels)

print(paste("Number of pixels above brightness threshold:", bright_pixel_count))
