# Supplementary material for: A novel antibody treatment reduces deformed wing virus loads in the western honey bee (Apis mellifera)
Source: mSphere. 2024 Oct 30;9(11):e00497-24. doi: 10.1128/msphere.00497-24 (PMC11580425; doi:10.1128/msphere.00497-24)
Supplement: Legend — qPCR data set legend. [file msphere.00497-24-s0002.docx]

qPCR dataset legend

Raw qPCR data for determining DWV levels in bees treated with the high dose (5.0% anti-DWV IgY (v/v)), low dose (0.5% anti-DWV IgY (v/v)), and the no IgY control. DWV Ct is the raw Ct values in reactions when using the DWV primers and the Ref Ct the raw Ct values when using the *Ndufa8* primers.
